# The Coexistence of Tinnitus and Temporomandibular Disorder: A Narrative Review on the Importance of an Interdisciplinary Approach

**DOI:** 10.3390/jcm13237346

**Published:** 2024-12-02

**Authors:** Klara Saczuk, Wiktoria Kal, Aleksandra Kaczała, Jędrzej Wawrzeń, Marzena Mielczarek, Tan Fırat Eyüboğlu, Mutlu Özcan, Monika Lukomska-Szymanska

**Affiliations:** 1 Department of General Dentistry, Medical University of Lodz, 251 Pomorska St., 92-213 Lodz, Poland; klara.saczuk@umed.lodz.pl; 2 Doctor of Dental Medicine Program, Medical University of Lodz, 251 Pomorska St., 92-213 Lodz, Poland; wiktoria.kal@gmail.com (W.K.); aleksandrakaczala@gmail.com (A.K.); jedrula31@gmail.com (J.W.); 3 Department of Otolaryngology, Laryngological Oncology, Audiology and Phoniatrics, Medical University of Lodz, 113 Żeromskiego St., 90-549 Lodz, Poland; marzena.mielczarek@umed.lodz.pl; 4 Department of Endodontics, Faculty of Dentistry, Medipol Unversity, Cibali Mah. Ataturk Bulv. No: 27, Fatih, Istanbul 34083, Türkiye; tfeyuboglu@yahoo.com; 5 Clinic of Masticatory Disorders and Dental Biomaterials, Center for Dental Medicine, University of Zurich, Plattenstrasse 11, 8032 Zurich, Switzerland; mutluozcan@hotmail.com

**Keywords:** somatosensory tinnitus, stomatognathic system, stress, temporomandibular disorders, temporomandibular joint, tinnitus, otologic symptoms

## Abstract

This review focuses on the coexistence of tinnitus and temporomandibular disorders in terms of epidemiological data, etiology, differential diagnosis, treatment, and interaction between the two disorders. PubMed, Google Scholar, and ClinicalKey digital databases were used to search for publications covering the years 2009–2024. Finally, 77 publications were used. The review followed recommendations of the Scale for the Assessment of Narrative Review Articles. The prevalence of tinnitus in individuals with TMD amounted to 2–59% and was reported as the main concomitant symptom. Several studies reported that tinnitus was eight times more common in TMD sufferers. Among patients with tinnitus, TMJ disorders were observed at a frequency of 19%, while in the group presenting with a high severity of the condition, TMJ disorders were observed more frequently (36%). Based on this review, in order to facilitate the diagnosis and future treatment of tinnitus and TMD patients, a proposal for a multidisciplinary diagnostic algorithm is presented in the article. The cooperation of an otolaryngologist, audiologist, neurologist, psychiatrist, dentist, and physiotherapist may be considered in clinical settings.

## 1. Introduction

Tinnitus is a phantom sound perception without corresponding external stimuli [1,2]. It can be any sound, e.g., hissing, ringing, humming, whistling, heard in the ear or head. The prevalence of this symptom differs between the studies, and it ranges between 5.1% to 42.7%, however, a range of 10–15% is the one most frequently reported in the literature [2,3]. Many individuals habituate to tinnitus, but around 1–2% of those affected experience lifestyle detriment, emotional difficulties, sleep deprivation, work hindrance, interference with social interaction, decreased overall health, and insecurity, together with depression, anxiety, and insomnia [2,4].

Tinnitus is a heterogeneous condition considering the cause, clinical characteristics, and accompanying symptoms. The main classification of this symptom includes subjective tinnitus, which is much more common, and objective tinnitus [5]. The first one is perceived only by the patient, and it can result from pathological changes in the entire auditory pathway. The second type can be heard by the examiner and the underlying source of pathological activity is located in the vascular or muscular systems (e.g., myoclonic contractions of the tensor tympani or altered blood flow in the blood vessels near the ear). Some other subtypings consider time from onset (acute and chronic tinnitus, below or above 6 months, respectively), the type of the perceived sound (tonal, noise-like tinnitus, multiple sounds), the presence of pulsing (pulsing and non-pulsing tinnitus), time of perception (temporary and permanent), and location (ear/s or head) [5,6]. Such a multidimensional diversity of tinnitus causes a lack of effective therapy that would work in each tinnitus patient [7,8]. Consequently, tinnitus remains an unmet diagnostic, therapeutic, economic, and social problem.

Commonly, tinnitus is thought to be an otological disorder, and in the majority of cases, cochlear sensorineural hearing loss plays a role [9]. One of the concepts explaining this phenomenon states that the phantom sound emerges as a result of compensatory events that occur after damage to cochlear hearing cells [10]. The impaired mobility of outer hearing cells results in the reduced auditory signal conveyed to the central auditory system. In the neurons of the dorsal and ventral cochlear nuclei, the inhibitory activity (reducing the release of inhibitory neurotransmitters including gamma-aminobutyric acid and glycine) is reduced, and simultaneously, the excitatory activity is raised by increasing excitatory neurotransmitters. As a consequence of these actions, the spontaneous firing rate grows and is further conveyed to inferior colliculi (IC) and then, via ascending fibers, to the medial geniculate body (MGB) of the thalamus. The spike in their spontaneous firing rate is consistent in spatial and temporal aspects. This sequence of events results in neuronal hypersynchrony and in consequence the neuroplastic changes at the level of the auditory cortex take place [11,12]. The peripheral processes lead to a reorganization of the tonotopic map of the auditory cortex. This results from deafferentation (in the course of cochlear hearing loss) and the decreased signal within the auditory pathway. This means that the neurons corresponding to a certain frequency of sound start to respond to the adjacent frequencies rather than responding to their primary frequencies, thereby reorganizing and extending the tonotopic map (tonotopic reorganization) [13]. Hence, the hyperexcitability in terms of spontaneous neuronal firing in the resting state, abnormal neural synchrony, and tonotopic reorganization in the auditory cortex are hypothesized to be major factors contributing to tinnitus generation and perception [14,15,16,17,18]. There are specific otological conditions that can be a cause of tinnitus: noise-induced hearing loss, acoustic trauma, age-related hearing loss (presbycusis), otosclerosis, sudden hearing loss, Meniere’s syndrome, vestibular schwannoma (acoustic neuroma), ototoxic cochlear damage, inflammatory condition of the external or the middle ear, and tympanic membrane perforation [19,20].

When the input from the somatosensory system can elicit or modify tinnitus, a special type of subjective tinnitus is considered which is called somatic (somatosensory) tinnitus (ST) [3,21]. The possible sources of somatosensory influence on the auditory pathway are the temporomandibular and upper cervical spine regions. The neural connections between these two systems have recently been described based on the animal model, but the basis for the clinical correlations between the ear symptoms and temporomandibular joint (TMJ) and masticatory system disorders was described by Costen in 1934 [21,22,23,24]. Hence, until animal research demonstrated the anatomical and functional connections between the auditory and somatosensory systems, the relations between tinnitus and temporomandibular disorders (TMD) were not straightforward. It remains unclear whether they coexist, depend on each other, or are unrelated.

The contemporary model of somatic tinnitus showed the connections between the dorsal cochlear nucleus (DCN) and the somatosensory brainstem nuclei receiving afferent information from the temporomandibular and upper cervical spine regions [21,22]. Therefore, DCN acts as the site of multi-sensory integration [25]. As a consequence of these anatomical and functional links, the abnormal somatosensory information can lead to an abnormal, mostly increased spontaneous firing rate in the DCN and, thus, to disturbed neuronal synchrony [26]. Therefore, when the input from the TMJ or cervical spine influences neuronal activity, it can evoke tinnitus or modify its physiological correlates [23,27]. The increased activation of the auditory brainstem nuclei during active protrusion in patients with tinnitus who could modulate tinnitus by jaw protrusion confirmed on the animal model the described connections between the dorsal cochlear nuclei and the temporomandibular somatosensory region [28].

Since tinnitus is primary regarded as a symptom of the ear or the auditory system, the role of the dentists or maxillofacial surgeons remains, at least initially, not obvious. This is especially because the patients themselves do not connect sounds experienced in the ear(s) with the disorders of the temporomandibular region or stomatognathic system. The stomatognathic system (SS) is a functional unit characterized by several structures: skeletal components (maxilla and mandible), dental arches, soft tissues (salivary glands, nervous and vascular supplies), and the TMJ and masticatory muscles. These structures act in harmony to perform different functional tasks (to speak, to break food down into small pieces, and to swallow) [29].

Moreover, the specialists themselves (dentists, maxillofacial surgeons, laryngologists, audiologists) may neglect this diagnostic and therapeutic issue due to the lack of interprofessional knowledge or cooperation. Even in the guidelines, the issue of involvement of the dentists in the diagnostics of somatic tinnitus due to stomatognathic system pathologies with suitable therapy seem underappreciated or neglected [30,31,32].

Despite the existence of many theories, the etiology of ST is not fully understood and is, therefore, a topic for further research and discussion. Theories and research findings point to a link between ST and TMD.

Thus, the aim of the study was to provide a comprehensive contemporary review of the coexistence of TMD and tinnitus and, based on the literature and clinical practice, to give some references for diagnostic management from the perspective of the dentist and otolaryngologist.

## 2. Search Strategy

The review complied with the recommendations of the Scale for the Assessment of Narrative Review Articles (SANRA) [33]. An electronic search was carried out in the PubMed, Google Scholar, and ClinicalKey digital databases using the keywords related to the topic search and combining the keywords using “AND” and “OR”. The search strategy employed was as follows: (tinnitus) AND (Temporomandibular disorders); (tinnitus) AND (stress); (tinnitus) AND (etiology); (tinnitus) AND (diagnosis); (Temporomandibular disorders) AND (diagnosis); (Temporomandibular disorders) AND (stress), (Temporomandibular disorders) AND (etiology); (somatosensory tinnitus) AND (Temporomandibular disorders); (somatosensory tinnitus) AND (etiology); (somatosensory tinnitus) AND (diagnosis); (temporomandibular dysfunction); (stress); (bruxism). The review was extended to the articles from their references and selected books.

Initially, 30,621 articles were found. Sources covering the years 2009–2024 were used in the research. After the removal of duplicates, 2356 articles were found in digital databases. The inclusion and exclusion criteria for the articles are presented in Table 1.

## 3. Results

**Figure 1:**
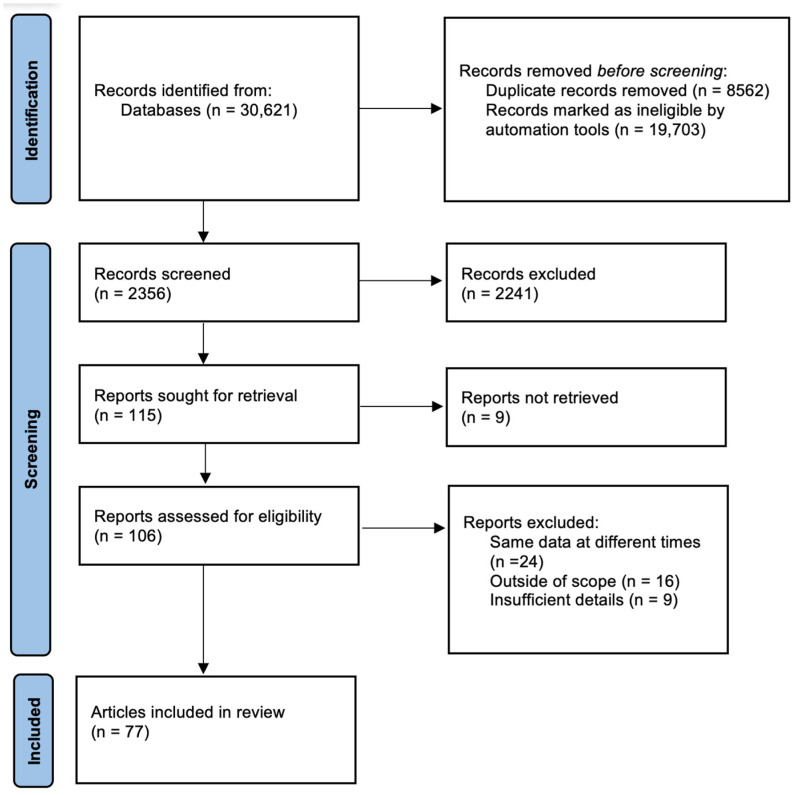
The PRISMA flow diagram.

Based on the inclusion criteria, 106 papers were further assessed for eligibility. Hence, fifty publications were rejected due to the lack of relevance to the objections chosen for this work. Most of the relevant papers were published within the last 7 years and the oldest one dealing with ST topics was published in 2009, indicating that this problem has been under investigation for a relatively short time [14]. Finally, 77 publications were qualified for this review. Both opinion and research papers were used, and the selected materials were in Polish and English. The PRISMA flow diagram presents the article assortment process (Figure 1).

### 3.1. Somatosensory Tinnitus

A subtype of subjective tinnitus is somatosensory tinnitus [3]. Usually, its etiology is unrelated to ear disorders [34]. In general, tinnitus is slightly more likely to occur in men, while ST is more frequently observed in younger women [3,35]. Tinnitus preceded by head or neck damage, or initiated simultaneously with complaints of pain in these areas, is one of the factors suggesting ST [34,36]. Symptoms such as frequent pain in the cervical spine, head or shoulder girdle, the presence of pressure-sensitive myofascial trigger points, increased tension in the suboccipital muscles and extensor muscles of the cervical spine, temporomandibular joint disorders, teeth clenching/bruxism, and dental disease may suggest ST [34,36,37].

One of the characteristics of somatic tinnitus is that it can be triggered or modulated by movements, somatic maneuvers, or pressure within the somatomotor system of the TMJ or cervical spine area [34,36,38,39]. Thus, the frequency, intensity, and location of the tinnitus may undergo temporary changes after being subjected to such factors [39]. This phenomenon is referred to as the somatosensory modulation of tinnitus [39]. The presence of this type of modulation may be a feature of ST, but on the other hand, it should be noted that somatosensory modulation is a phenomenon that also occurs in individuals without concomitant somatic disorders and in individuals with tinnitus of other origins [34,36,37,39]. The presence of somatosensory dysfunction is required to establish ST [37,39]. The ability to modulate tinnitus in ST patients can be managed in several ways. They include the movements of the head, neck, jaw, orofacial and eye movements, and less frequently limbs, fingers, pressure on the muscles of the head and neck, on myofascial trigger points, the cutaneous stimulation of the face, hands or fingertips, or the adoption of abnormal body posture [34,36,37,39,40]. The extent of these changes varies by individual, and due to the effect of varying muscle tone (e.g., of the neck) on noise, i.e., tinnitus modulation, they may cause periodic variations in tinnitus perception [37].

In 2017, three groups of criteria were identified that strongly suggest the presence of ST with the influence of the SS as a factor affecting tinnitus modulation (Table 2) [36]. Michiels et al. emphasizes that these criteria strongly suggest a diagnosis of ST, but they are not an absolute requirement for a diagnosis of ST [36].

### 3.2. Temporomandibular Disorders

TMD is a term that encompasses dysfunctions in the TMJ, the muscles of mastication, and the associated musculoskeletal structures of the head and neck, including the cervical spine [41,42]. TMD is the third most common pathology in the SS, after caries and periodontal pathologies, and it affects 15% of the general population, regardless of race [43,44,45]. Women are twice as likely to develop TMD, especially persistent TMD associated with pain symptoms [35,44,45,46,47,48,49]. Hormonal, biological, and psychosocial factors, as well as pain threshold, are all considered likely causes of this condition [43,50]. Women are frequently more motivated to seek treatment than men, which may be the reason why TMD is diagnosed more often in this group [44]. The prevalence of TMD increases with age, with the peak incidence occurring at 35–44 years of age [43,44,51]. TMD is more commonly encountered in young and middle-aged adults than in children and older adults [52]. Currently, the increase in TMD cases is also connected with working in home offices and consistent bad posture [2]. There has been a steady climb in the number of patients suffering from TMD in highly developed countries, and as a result, it is becoming progressively popular to determine it as a civilization disease [43,53]. The incidence of TMD is conditioned multifactorial, and this leads to the dynamic increase in these dysfunctions [42,45,52,54,55]. The following TMD risk factors are distinguished: biological, behavioral (sleep hygiene, ability to cope with stress), environmental (exposure to stressful situations), and emotional (greater sensitivity to stress in women) [31,42,52,54,56,57]. Comorbid factors may act synergistically and are divided into predisposing, initiating, and perpetuating factors [31,34,44,54]. Predisposing conditions involve those increasing the risk of TMD development. These are systemic, psychological (depression, anxiety, autoimmune diseases), structural (deviation in form, subluxation, spontaneous dislocation of articular disk), genetic (metabolism of catecholamines, adrenergic receptors), and the individual’s sensitivity to pain [42,45,54]. Initiating factors include the overloading of the TMJ (parafunction, tooth loss, ill-fitting dentures, unfixed missing teeth) and/or its trauma [54]. Moreover, muscular tension and/or metabolic disorders can harden the TMD and consequently hinder healing [54].

An important factor that can lead to the development of TMD is bruxism [43,58]. A new definition was established in 2018 at the International Consensus—Assessment of Bruxism Status. It distinguishes sleep bruxism as “masticatory muscle activity during sleep that is characterized as rhythmic (phasic) or non-rhythmic (tonic) and is not a movement disorder or a sleep disorder in otherwise healthy individuals” and awake bruxism defined as “masticatory muscle activity during wakefulness that is characterized by repetitive or sustained tooth contact and/or by bracing or thrusting of the mandible and is not a movement disorder in otherwise healthy individuals” [58]. Bruxism also leads to the abrasion of the incisal edges and occlusal surfaces of the teeth and even their loss, severe pain in the masticatory muscles, and changes in the periodontium [43]. It is worth noting that stress is one of the main etiological and aggravating factors of bruxism [59].

### 3.3. The TMD Classification

The TMD classification distinguishes temporomandibular joint disorders (TMJD), myofascial pain disorder (MPD), and cervical spine dysfunction (CSD) (Table 3) [38,42].

TMJD can include acoustic symptoms within the TMJ (crackling, crepitation), mandibular mobility disorders (locking, skipping, limited range of motion), TMJ area pain or degenerative changes in the TMJ [41,42,45,54,61,65]. The areas that trigger typical symptoms of MPD (spasms, pain, and skeletal muscle dysfunction) are the fascia and attachments of the affected muscles [42]. Masticatory muscle disorders are the result of TMJD [42]. On the other hand, CSD affects the vertebrae, ligaments, and muscles of the cervical spine [42]. The association of CSD with other TMD pain symptoms in the head is a consequence of neuronal connections between head and neck structures, the impaired action of the descending pathway of pain inhibition, and central sensitization [64]. Based on the diagnostic criteria for TMD (DC/TMD), the described disorders can also be divided into pain-related, intra-articular TMJ disorders and complex symptoms [44,45]. Pain-related disorders can involve muscles, joints or the head and are experienced during chewing and can be modified by functional or parafunctional movements of the mandible, during muscle and/or TMJ area palpation [45,60]. Among the complaints that may coexist in TMD patients there are tinnitus, fibromyalgia, irritable bowel syndrome, chronic fatigue syndrome, and depression [44,53]. Their presence is also one of the predisposing factors for the development of TMD [45]. Other possible concomitant symptoms present in TMD patients are headache secondary to TMD, orofacial pain, and otologic symptoms (Table 4). TMD patients may also experience sleep disorders which are again one of the predisposing factors for the development of TMD [45,66]. However, it is worth noting that to arrive at a clinical diagnosis of TMD, a thorough head and neck examination must be concluded, in which a clinical otological condition such as otitis or another possible cause of ear pain and tinnitus is excluded. Symptoms that may occur if the patient is diagnosed with TMD are presented in Table 3 and Table 4.

### 3.4. Theories Linking Tinnitus with Temporomandibular Disorders

There are many theories explaining the relationship between TMD and otologic disorders. These theories focus on the available knowledge about the anatomical structure of the head and neck area and the physiological and pathophysiological processes within these structures.

#### 3.4.1. Joint Phylogenetic and Ontogenetic Development

Among the hypotheses explaining the relationship between TMD and oto-vestibular disorders (ear pain, tinnitus, dizziness, sudden hearing impairment), phylogenetic and ontogenetic development are also mentioned [54,70,71]. The medial pterygoid muscle, tensor veli palatini muscle, and tensor tympani muscle develop from the first pharyngeal arch [71]. Parallel embryonic SS and ear development are explained by the shared innervation and vascularization of these structures [54].

#### 3.4.2. Topographical Proximity

The topographical proximity of the SS and the ear is among the considered anatomical relationships [54,70,72]. Due to the topographical proximity of the TMJ and the tympanum, there is the possibility (indirectly through the discomalleolar ligament and directly through the pressure caused by the mandibular condyle) of the stimulation of the auriculotemporal nerve, resulting in a contraction of the tensor tympani muscle [71]. Dysfunction involving the posterior displacement of the mandibular condyle in the TMJ can lead to chronic injury to the chorda tympani, a branch of the facial nerve, resulting in a contraction of the stapedius muscle which entails an immobilization of the stapes, thus hearing impairment and tinnitus [53].

#### 3.4.3. Joint Sensory Innervation

**Figure 2:**
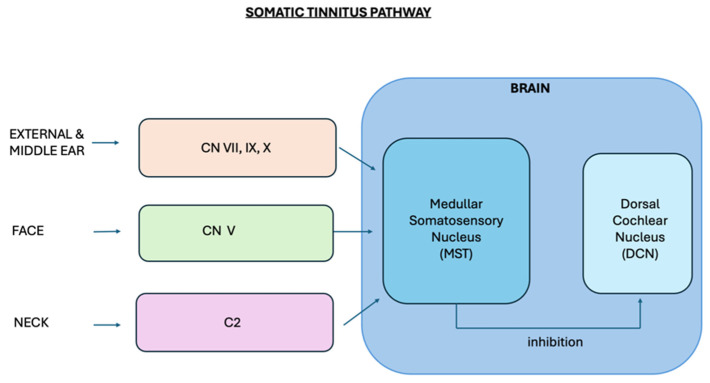
Somatic tinnitus pathway.

The involvement of cranial nerves—trigeminal nerve (V), facial nerve (VII), glossopharyngeal nerve (IX), vagus nerve (X)—the cervical plexus—C2 and C3—and also the sympathetic fibers of the internal carotid plexus in the transmission of ailments between the SS and ear areas was considered (Figure 2) [70]. The given nerve structures are responsible for the sensory innervation of the ear, while the main sensory nerve of the face is the V cranial nerve [73]. The spinal nucleus of the trigeminal nerve receives signals from mechanoreceptors, thermoreceptors, and nociceptors of the face, which are further conducted by the above-mentioned structures of the nervous system.

#### 3.4.4. Joint Motor Innervation

The trigeminal nerve gives motor innervation to the muscles of mastication [73]. The third branch of this nerve—the mandibular nerve—innervates masseter muscles together with the tensor tympani muscle [70,71,74,75]. Often, an increase in muscle tension in one group is accompanied by excessive tension in the other muscle group [71]. Excessive activity of the masticatory muscles can lead to a dysfunction of the tensor tympani muscle and this can cause tinnitus as a result of the pathological mechanisms described earlier (see Section 3.4.2) [53,71].

#### 3.4.5. Neuromodulation

The theory explaining the possible influence of stimuli arriving from the head and neck area on the formation or modulation of ST perception is based on the phenomenon of neuromodulation [35]. Stimuli received by the sensory receptors are transmitted to specialized areas of the sensory cortex [35]. The connections between sensory neurons from the SS region and the areas of the sensory cortex are associated with hearing and sound analysis [53,72]. The anatomy indicates the presence of multimodal functional interaction between areas of the sensory cortex, enabling a faster perception of information from the external environment [35]. When the sensory representation of a particular area of the sensory cortex is lost, compensation from other sensory modalities occurs. This phenomenon is called cross-modal plasticity [35]. Most likely, due to neuroplasticity, the abnormal interactions between sensory modalities, somatomotor systems, and neurocognitive and neuro-emotional networks can contribute to the development of ST [38]. As a result of unilateral somatic damage, tinnitus develops in the ipsilateral ear [38].

#### 3.4.6. Stress

Stress is the response of the body to factors that can realistically or only seemingly threaten homeostasis [54,55]. Numerous studies show an association between stress and the occurrence of TMD symptoms [43,47,53,76,77,78,79]. Increased and chronic stress can lead to tissue damage and the development of an excessive increase in muscle excitability, including mastication muscles, can evoke or exacerbate other diseases including TMD and tinnitus, and can increase the severity of symptoms of these disorders [34,45,54,76,77,78,80,81,82]. Stress also results in an imbalance of serotonin and catecholamines, resulting in pain [47]. Behavioral and psychological factors are considered the most significant group of etiological factors of TMD [46]. The involvement of stress in the occurrence of tinnitus is supported by the fact that the first symptoms may appear during or after a period of intense stress. However, conscious avoidance and the elimination of stressful factors have been found to reduce the risk of TMD pain and intra-articular symptoms and to diminish the severity of subjective tinnitus [44,76,83,84,85,86]. These facts may support the role of stress in the etiology of both disorders.

### 3.5. Clinical Data

Clinical studies indicate the frequent simultaneous occurrence of tinnitus and TMD, prompting the search for an interaction between the two disorders [46,77]. The prevalence of tinnitus in individuals with TMD is reported to be the the main concomitant symptom which amounted to 2–59% [77]. Severeal studies reported that tinnitus was 8 times more common in TMD sufferers [37,76,77,78]. Among patients with tinnitus, TMJ disorders were observed at a frequency of 19%, while in the group presenting with high severity of the condition, TMJ disorders were observed more frequently (36%) [35]. In most cases, the presence of ST is not accompanied by other ear disorders [34]. Symptoms from the SS and cervical spine area may occur simultaneously with the presence of ST [34,36]. Triggering or modulation of tinnitus due to movements, pressure or somatic maneuvers of the structures of TMJ or cervical region is also possible [34,36]. A decrease in tinnitus intensity was observed as a result of head and neck maneuvers, while this effect is rarely achieved by maneuvers within the TMJ itself, namely maneuvers in the TMJ most often result in an increase in tinnitus [37]. Daily changes in neck muscle tension can cause modulation of tinnitus perception, depending on the time of the day [37]. The somatic region which is the most frequent source of tinnitus modulation is the TMJ area [36]. In the case of TMD, ear pain was the most common, followed by the others: tinnitus, a feeling of fullness in the ear, dizziness, and subjective hearing loss [46,73]. In patients with concomitant TMD and oto-vestibular disorders, ear pain was reported to occur at a frequency of 50%, and tinnitus in 45.5% of the cases [53,72]. However, another study carried out by Porto de Toledo and colleagues proved that tinnitus prevalence was 52.1% [52]. Among tinnitus patients, various TMD symptoms were present [46]. The most commonly reported problem was masticatory muscle pain, while in 33% of cases acoustic symptoms in TMJ, or masticatory muscle fatigue [46]. In clinical studies reporting simultaneous unilateral TMD and unilateral otologic symptoms, ipsilateral localization of symptoms was significantly more common [42,77].

### 3.6. Therapeutic Strategies

Physical therapy is typically regarded as the most effective treatment for symptoms associated with TMD and somatic tinnitus [87]. According to studies, a multimodal therapy that targets the TMJ, cervical, and masticatory muscles includes exercises, manual therapy, physical therapy, an acrylic splint and patient education [82,83,88,89]. Cervico-mandibular manual treatment specifically improved the patient’s physical (by increasing mandibular active range of motion), psychological (by reducing depressive symptoms), and clinical (by relieving impairment linked to tinnitus and TMD) [90]. Both patient groups experienced localized hypoalgesia, although the manual therapy group experienced it more than the other, as indicated by an increase in the masticatory muscles’ pressure pain thresholds (PPTs) [90,91]. These results support the neuro-physiological advantages of exercise and manual therapy for the central nervous system.

With tailored strategies for each patient’s unique tinnitus subtype and characteristics, numerous interdisciplinary conservative treatments have demonstrated a positive effect on tinnitus control throughout the years [91,92,93]. Behavioral and educational approaches, along with congnitive behavioral therapy were among the multimodal treatments that seemed to be more successful than a single treatment [89,94,95]. The need for more study with an efficient methodological approach was brought to light, nevertheless, by the use of non-homogeneous diagnostic criteria, a lack of knowledge about the medical conditions of the patients, and inadequate research methodology with limited poor validity of the available data [52].

## 4. Summary

Typically, tinnitus patients are initially referred to an otolaryngologist or neurologist. This is justified since the basis of the diagnosis of tinnitus is the performance of examinations of the ear, as well as the central nervous system [39,53]. Despite the emergence of standardized diagnostic criteria for ST, it can also be diagnosed regardless of the presence or absence of the symptoms listed [36]. In view of such great heterogeneity in the etiology and characteristics of tinnitus, an interdisciplinary diagnostic approach is necessary. Subsequently, depending on its outcomes, interdisciplinary therapeutic management may be required [2,56]. Pure tone audiometry is the primary examination performed in a patient suffering from tinnitus, and ST can be indicated by the presence of bilaterally symmetrical hearing thresholds—both normal and abnormal, with concomitant unilateral or asymmetric tinnitus [36,39,40]. Discrepancy between the lateralization of unilateral TMD and unilateral tinnitus (which are then on the opposite sides) is considered to exclud the existence of correlation between TMD and tinnitus in a given patient [77]. According to the literature, in more than 50% of cases, the cause of pain in the ear area is not located in the ear [72]. Diagnostics, which do not include TMD as a possible etiological factor of otologic symptoms, may be a cause of ineffective treatment in that group of patients. A set of following criteria that provide strong evidence for the diagnosis of somatic tinnitus was developed: the ability to modulate tinnitus by voluntary movements of the head, jaw, neck, or eyes, by the somatic maneuvers and/or by the pressure on myofascial trigger points; cervical, head, and shoulder pain which coexist or aggravate simultaneously with tinnitus; tinnitus preceded by a head or neck trauma or increases on bad posture; increased tension of the cervical spine’s suboccipital and extensor muscles; temporomandibular disorders (TMD) in conjunction with tinnitus; and/or clenching, bruxism, and dental disease. [36,96]. People reporting tinnitus are three times more likely to also report myofascial pain that those without it, while the correlation between the lateralization of tinnitus (or its increase when bilateral) with the side of the body that is in pain is 54% [97,98]. A further factor distinguishing between somatic and “otologic” tinnitus is when the audiogram does not account for unilateral tinnitus. Furthermore, a study published suggests that there is a strong association between joint pathology and otologic symptoms, especially tinnitus, and this relationship exacerbates as joint pathology progresses. According to these data, there may be a particular subtype of tinnitus known as “TMD-related somatosensory tinnitus” [99]. The specific features of tinnitus which should be attributed to somatic TMJ tinnitus are listed as strong evidence for such tinnitus origin.

**Figure 3:**
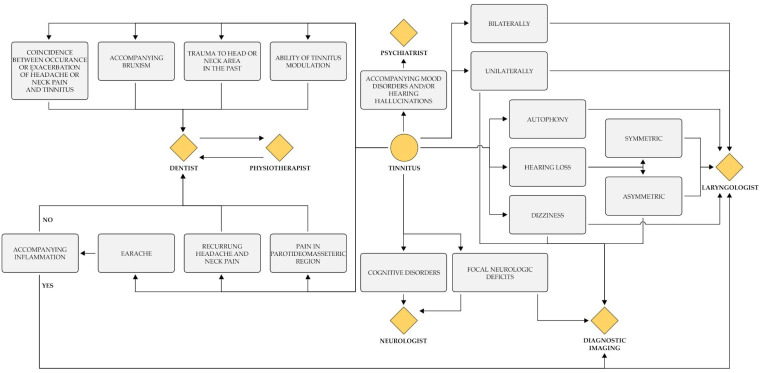
Procedural algorithm in case of selected otological and neurological symptoms.

When otolaryngological examination together with imaging do not point to possible etiology of the audiovestibular symptoms (ear pain, tinnitus, dizziness, and sudden hearing impairment), other disorders of the head and neck from the area of the interest of the dentist or physiotherapist should be considered According to the studied literature reduction in the severity of tinnitus as a result of treatment of SS disorders was advocated [52,77] while tinnitus resolution or improvement in 43%–86% after TMD therapy was described [52]. Hence, the knowledge of the etiology and therapy of ST should be expanded in the community of both dentists and otolaryngologists. Moreover, proper diagnosis and further treatment require a multidisciplinary team properly trained in this field. Consequently, the cooperation between the otolaryngologist, audiologist, neurologist, psychiatrist and the dentist, and physiotherapist should be considered in clinical settings [39,52]. It is also significant to educate patients about the etiology of tinnitus, which would help them reach the right professionalist and thus the right treatment. Based on this review, in order to facilitate the diagnosis and future treatment of tinnitus and TMD patients a proposal for a multidisciplinary diagnostic algorithm is presented in Figure 3. Furthermore, in complex cases, more than one consultation should be considered to take full advantage of diagnostic possibilities from different medical fields.

Given the importance of behavioral and psychological factors in the development of TMD and the role of stress in the development of tinnitus and exacerbation of the symptoms of these pathologies, stress should not be overlooked in the discussion of these two ailments [35,47,48,56,77,83]. In fact, conscious avoidance and the elimination of stress factors reduce the risk of TMD pain and intra-articular symptoms and diminish the severity of subjective tinnitus, improving patients’ quality of life [44,76,84,85]. The fact that the first symptoms of tinnitus appear during or after stress confirms its influence on this ailment [2]. It should be noted that the perception of tinnitus can itself be a stressor, contributing to increased physiological arousal and psychological distress (anxiety, depression, sleep and concentration disorders) [65,86,100]. Patients with severe tinnitus were more likely to suffer from psychiatric disorders than patients with mild tinnitus [76]. Based on the correlations discussed above, it is therefore possible to consider stress as a predisposing collective factor that can cause both TMD and tinnitus [53,70,72,73,77,101]. Studies confirm the possibility of a positive effect of lowering stress levels on reducing the severity of TMD and tinnitus symptoms [44,76,77,84,102]. The hypothesis of the existence of a correlation between TMD and tinnitus is supported by the positive effect of TMD therapy on reducing the severity of tinnitus reported in some patients [77].

Based on the anatomical and functional connections discussed, theories are being developed to explain the relationship between the coexistence of TMD and tinnitus. It is anticipated that the neuromuscular dysfunction of the masticatory muscles leads to a disturbance in the conduction of sound signals through the ear as a result of irritation of the tensor veli palatini and tensor tympani muscles [53,72]. Otological complaints can be explained by spasms of the blood vessels supplying the cochlea [53,72]. This spasm would be produced by a disturbance in the activity of the trigeminal nerve [72]. The co-occurrence of oto-vestibular symptoms and TMD can be associated with the fact of the common phylogenetic and ontogenetic development of the medial pterygoid, tensor veli palatini, and tensor tympani muscles [70]. The phenomenon of modulation of tinnitus perception is not yet fully understood, but there is scientific evidence supporting the presence of neural connections between the somatosensory and auditory systems [39].

Dentists must become more engaged in exploring this seemingly non-stomatological topic and increase awareness among otolaryngologists about the possible effective collaboration with dentists. The awareness of the possible link between tinnitus and TMD is crucial for a proper diagnosis and, consequently, an appropriate treatment plan.

## 5. Conclusions and Future Perspectives

The relatively small number of articles exploring ST and TMD simultaneously demonstrated the importance of collecting and organizing the available information. Epidemiological studies confirm a higher prevalence of tinnitus in individuals with TMD than in the general population, and stress presents itself as a possible etiological and aggravating factor for both conditions. Among otolaryngologists and dentists, it is crucial to be aware of the potential link between these disorders in order to improve the effectiveness of diagnosis and treatment. Also, the comprehensive diagnostics of tinnitus should include otolaryngological and audiological evaluation, an assessment of SS function, the medical imaging of specific areas of the head and neck, and psychological examination. Creating interdisciplinary research and therapeutic groups would be beneficial, enabling a holistic management of tinnitus coexisting with TMD. The multidisciplinary diagnostic and therapeutic management would improve the development of clinics or hospital departments specializing in tinnitus with a focus on ST coexisting with TMD. Based on the findings of this review, it is unclear however whether concurrent TMD and tinnitus coexist independently or are related, which needs to be further investigated.

## Figures and Tables

**Table 1:** The inclusion and exclusion criteria for articles.

Inclusion Criteria	Exclusion Criteria
Research and articles on tinnitus	Articles without full text availability
Research and articles on somatosensory tinnitus	Articles published before 2000
Research and articles on temporomandibular disorders	Same data that were published at different times
Research and articles including stress, bruxism	Articles in a language other than English or Polish

**Table 2:** Criteria indicating somatosensory tinnitus.

Criteria	Indicators Suggesting ST Diagnosis
Perception	Tinnitus pitch, loudness and/or location vary [35]
	Tinnitus can be modulated [34,36]
Tinnitus modulators	Voluntary movements of the head, neck, jaw or eyes [34,36]
	Somatic maneuvers [34,36]
	Pressure on myofascial trigger points [34,36]
Accompanying symptoms	Tinnitus and neck, head or shoulder girdle pain appear simultaneously [34,36]
	Frequent head or neck complaints [34,36]
	Tinnitus preceded by head or neck trauma [34,36]
	Increased suboccipital muscles tension [34,36]
	Increased tension of extensor muscles of the cervical spine [34,36]
	Intensified tinnitus during bad postures [34,36]
	Presence of active (pressure sensitive) myofascial trigger points [34,36]
	TMJ disorders [34,36]
	Teeth clenching/bruxism [36,41]
	Dental diseases [36,41]

**Table 3:** Classification of TMD.

Diagnosis			Localization	History	Examination
M P D	Muscle pain [45,60]	Local myalgia	Pain local to palpation	At least 2 of the following symptoms: Pain modified with jaw movement (function or parafunction) Jaw pain Pain in temple Pain in ear Pain in front of ear	Masseter and temporal muscle pain on palpation and/or during assisted or unassisted jaw movement [45,60]
		Myofascial pain	Muscle and fascia		
		Myofascial pain with referral	Pain spreading outside the body of the muscle		
	Arthralgia [45,60]		TMJ region		TMJ pain on palpation and/or during assisted or unassisted jaw movement
TMD	Intra-articular disorders	Articular disk displacements	TMJ region	Reduced mouth opening inability to eat or eating disturbance [61]	Presence of crepitus, clicking, jumping in TMJ during mandible movements: abduction, adduction, lateral movement in maximum mandible abduction (correct < 40 mm)
		Degenerative joint disease		Crepitus and little pain in TMJ during every jaw movement	Presence of acoustic symptoms (crepitus) in TMJ during mandible movements: abduction, adduction, lateral movements
		Subluxation		Impossible mandible adduction, manipulation is necessary to close the mouth [62]	Pain and discomfort in the joints and masticatory muscles, clicking sound can be a sign of subluxation [62]
C S D	Limited mobility of cervical spine [42,60]		Cervical spine	Neck and shoulders pain [63]	Evaluation of the following: Craniocervical posture Range of cervical spine movement (bend, rotation) [60] Cervical muscle–palpation [64]
	Poor efficiency of the deep cervical flexor muscles [60]				Evaluation of range of motion of cervical spine (bend, rotation) [60]

**Table 4:** Possible concomitant symptoms of TMD.

Symptom	Characteristics	Etiology		Location
Otological symptoms associated with TMD [42,53]	Sound hypersensitivity [53] A feeling of ear fullness [53] Tinnitus longer than 5 min [53] Otalgia [53] Dizziness [53] Deterioration of hearing [53] Acute and piercing pain during mandible movement [42] Above symptoms appear as unilateral and ipsilateral to affected joint [42]	Musculoskeletal background [42] TMJ disorders [42,67] Abnormal maxilla growth causing Eustachian tube malfunctions [42] Common embryological origin of middle ear ossicles and mandible [67] Inflammatory processes of the cervical spine joints or TMJ [67]		Ear area [42]
Headache secondary to TMD [64,68]	Blunt and severe pain [42] May lead to chronic course of pain [67] Remission and exacerbation periods are present [42] Pain is modified by mandible movement (physiological and/or para-functional) [42,54] or by pressure on the TMJ or masticatory muscles [67] Headaches correlated with TMD manifestation [64,68] Headache exacerbation along with TMD progress [64,68] Reduction or resolution of headaches simultaneously to reduction or resolution of TMD [67]	Disorder involving structures in the temporomandibular area [67] Increased activity of muscles or disorders of TMJ [42,67] Morning headaches due to bruxism during sleep and/or sleep disorders [42]		Postural muscles [42] Masticatory muscles [42] Temporal area [41,45,60] Ipsilaterally to TMJ [67] Bilaterally if muscles are involved [67]
Orofacial pain [42,69]	Appears after meals as feeling of heaviness and fatigue of masticatory muscles [42] Any pain related to soft or hard tissues of oral cavity or face [68,69] Pain intensity varies from gentle to severe [68,69]	Musculoskeletal background [42] Trigger points or muscle fatigue (awake bruxism, severe or chronic pain, reduction in occlusion related to posterior teeth loss) [42] Reduction in blood flow and accumulation of metabolites in muscle tissue [68,69]	Masseter muscle [41]	Mandible area [69]
			Temporal muscle [41]	Head area [68,69]
			Medial pterygoid muscle [41]	Throat (pain upon swallowing) [68,69] Mandible angle area [68,69]
			Lateral pterygoid muscle [41]	Ear area [68,69]

TMD—temporomandibular disorders; TMJ—temporomandibular joint.

## Data Availability

Not applicable.
